# Impact of HIV infection and integrase strand transfer inhibitors-based treatment on the gut virome

**DOI:** 10.1038/s41598-022-25979-5

**Published:** 2022-12-15

**Authors:** Pablo Villoslada-Blanco, Patricia Pérez-Matute, María Íñiguez, Emma Recio-Fernández, Daan Jansen, Lander De Coninck, Lila Close, Pilar Blanco-Navarrete, Luis Metola, Valvanera Ibarra, Jorge Alba, Jelle Matthijnssens, José A. Oteo

**Affiliations:** 1 Infectious Diseases, Microbiota and Metabolism Unit, Infectious Diseases Department, Center for Biomedical Research of La Rioja (CIBIR), C/Piqueras 98, CIBIR Building, Third Floor, 26006 Logroño, La Rioja Spain; 2 Laboratory of Viral Metagenomics, Department of Microbiology, Immunology and Transplantation, Rega Institute, KU Leuven, Leuven, Belgium; 3 Centro de Salud Siete Infantes de Lara, Logroño, La Rioja Spain; 4 Infectious Diseases Department, Hospital Universitario San Pedro, Logroño, La Rioja Spain

**Keywords:** Computational biology and bioinformatics, Microbiology, Diseases, Pathogenesis

## Abstract

Viruses are the most abundant components of the human gut microbiome with a significant impact on health and disease. The effects of human immunodeficiency virus (HIV) infection on gut virome has been scarcely analysed. Several studies suggested that integrase strand transfers inhibitors (INSTIs) are associated with a healthier gut. Thus, the objective of this work was to evaluate the effects of HIV infection and INSTIs on gut virome composition. 26 non-HIV-infected volunteers, 15 naive HIV-infected patients and 15 INSTIs-treated HIV-infected patients were recruited and their gut virome composition was analysed using shotgun sequencing. Bacteriophages were the most abundant and diverse viruses present in gut. HIV infection was accompanied by a decrease in phage richness which was reverted after INSTIs-based treatment. β-diversity of phages revealed that samples from HIV-infected patients clustered separately from those belonging to the control group. Differential abundant analysis showed an increase in phages belonging to Caudoviricetes class in the naive group and a decrease of Malgrandaviricetes class phages in the INSTIs-treated group compared to the control group. Besides, it was observed that INSTIs-based treatment was not able to reverse the increase of lysogenic phages associated with HIV infection or to modify the decrease observed on the relative abundance of Proteobacteria-infecting phages. Our study describes for the first time the impact of HIV and INSTIs on gut virome and demonstrates that INSTIs-based treatments are able to partially restore gut dysbiosis at the viral level, which opens several opportunities for new studies focused on microbiota-based therapies.

## Introduction

Human immunodeficiency virus (HIV) infection is now considered a chronic disease related to a set of structural and functionality changes of the gut epithelial barrier followed by modifications in the composition and functionality of gut microbiota (GM). These changes are not completely restored despite the use of antiretroviral treatments (ARTs)^1^. Most of the current microbiome studies have concentrated and analysed the effects of HIV infection and different ARTs on the bacterial component of GM^2–8^. However, GM consists not only of bacteria but also of viruses, archaea, fungi, and other eukaryotic organisms. In this context, it is worth mentioning that viruses are the most abundant components of the gut microbiome in human beings with a significant impact on health, being an emerging field of study^9^. Thus, bacteriophages can perturb the bacterial community and indirectly influence gut health and interact with the human immune system^10,11^. However, the importance of gut virome has been understudied. Only few studies have revealed an altered gut viral composition in different pathological conditions such as cancer^12,13^, type I diabetes^14^ and inflammatory bowel disease (IBD)^15,16^. Little is known about the impact of HIV infection on gut virome (bacteriophages and eukaryotic viruses). Most of the few studies published in this field are focused on plasma^17–20^, semen^21^, saliva^22^, and cervix^23^ communities and there is only one study that has evaluated the effects of HIV infection on gut virome, although only DNA viruses have been assessed in such study^24^.

Several studies suggest that not all ARTs exert similar effects on the gut bacteriome. In fact, our group has demonstrated that ARTs based on INSTIs, key components of ART in the treatment of naive HIV-infected patients^25–27^, were associated with levels of systemic inflammation, soluble CD14 (sCD14) plasma levels, and microbial diversity similar to those observed in uninfected controls, suggesting a healthier gut and potentially fewer HIV-related complications^28,29^. However, to our knowledge, there are no studies focused on the effects of the actual ART regimens based on INSTIs on the gut virome and its physiological relevance in terms of health. Thus, the objective of this work was to analyse the associations between HIV infection and INSTIs-based therapies in first line of treatment on gut virome composition (including both DNA and RNA viruses).

## Methods

### Patient recruitment

HIV-infected patients (naive and under ART) were recruited from the Infectious Diseases Department at Hospital Universitario San Pedro (HUSP) (Logroño, Spain) from March 2019 to February 2021. Patients recruited during COVID pandemic did not report any symptoms related to COVID before sample collection and were not vaccinated in the previous month of the enrolment. The group of ART-treated patients included HIV-infected patients in first line of treatment with INSTIs (dolutegravir or bictegravir) to avoid confounding effects due to previous treatments. These patients were on ART for at least one year and presented a viral load < 20 copies/ml. Along with the INSTIs, the backbone of the treatment was one or two Nucleoside Reverse Transcriptase Inhibitors (NRTIs). Thus, 10 out of 15 INSTIs-treated patients (66.67%) were treated with dolutegravir as INSTI and abacavir/lamivudine as backbone, 2 out of 15 (13.33%) were treated with dolutegravir as INSTI and lamivudine as backbone and 3 out of 15 (20%) were treated with bictegravir as INSTI and emtricitabine/tenofovir alafenamide as backbone (Supplementary Table 1). All HIV-infected ART-treated patients were immune responders, which means that their CD4+ T-cell levels reached at least 200 cells/µl after treatment. On the other hand, the group of naive patients (patients recently diagnosed and without treatment) was recruited after the same day that were diagnosed as HIV-positive. The presence of acquired immunodeficiency syndrome (AIDS), mode of transmission, sexual preference, and coinfection with hepatitis B virus (HBV) and/or hepatitis C virus (HCV) were registered. In case of coinfection, degree of liver fibrosis was evaluated by FibroScan^®^ (Echosens, Paris, France) method. Patients were classified according to the METAVIR scoring system (F0, no fibrosis; F1, portal fibrosis without septa; F2, portal fibrosis and few septa; F3, numerous septa without cirrhosis; F4, cirrhosis)^30^. CD4+ T-cell, CD8+ T-cell counts, and viral load were measured using flow cytometry (NAVIOS EX, Beckman Coulter) and COBAS 6800 Analyzer (Roche Molecular Systems Inc., Branchburg, New Jersey, USA), respectively, as a clinical procedure in the HUSP. Healthy patients (non-HIV-infected patients) were also recruited as control group (n = 26). For both HIV-infected patients and controls, following exclusion criteria were applied: < 18 years, patients who do not sign the informed consent, pregnant women (to avoid confounding effects^31^), individuals with inflammatory disease in the last 2 months, patients treated with antibiotics, anti-inflammatory drugs, immunosuppressive drugs, statins or probiotics in the last 2 months, individuals with renal insufficiency, patients with neoplasms, individuals with history of intestinal surgery (except from appendectomy or cholecystectomy), IBD, celiac disease, chronic pancreatitis or any other syndrome related to intestinal malabsorption^28^. Patients treated with statins were excluded because it was demonstrated that this therapy can cause gut dysbiosis^32,33^. Finally, weight, height, waist circumference, systolic and diastolic pressure, alcohol consumption, and smoking habits were also registered from all participants. Supplementary Fig. 1 shows a flowchart of patient recruitment.

### DNA and RNA extraction from stool samples and shotgun sequencing

Fresh stool samples were received at CIBIR, collected, aliquoted in tubes with O-ring caps (45-55 mg) and stored at −80 °C for further analysis. These samples were also processed for the bacteriome analyses that has already been published^29^. In addition, stool samples were thawed and fecal viral DNA and RNA were extracted using the NetoVIR protocol as described before^34^. The aliquots were suspended in sterile dPBS (10%) and homogenized using the MINILYS homogenizer (Bertin Technologies) for 1 min at 3000 rpm. Homogenates were centrifuged for 3 min at 17,000*g* and filtered using a 0.8 µm PES filter (Sartorius). Filtrates were treated with micrococcal nuclease (New England Biolabs) and benzonase (Novagen) at 37 °C for 2 h. Viral nucleic acids were extracted using the QIAMP^®^ Viral RNA mini kit (Qiagen, Venlo, Netherlands) without addition of carrier RNA to the lysis buffer. Subsequently, random amplification was performed using a modified version of the WTA2 kit (Sigma-Aldrich) with the following parameters: 94 °C for 2 min, 17 cycles of 94 °C for 30 s and 70 °C for 5 min. The WTA2 products were purified using the MSB Spin PCRapace kit (Stratec Molecular). Quantification of purified product was performed using Qubit™ dsDNA HS Assay kit with the use of a Qubit 2.0 fluorometer (Thermo Fisher Scientific, MA, USA). Sequencing libraries were prepared using the Nextera XT DNA Library kit (Illumina). Sizes of the libraries were checked with the Bioanalyzer 2100 using the High Sensitivity DNA kit (Agilent Technologies, USA).

Sequencing was performed using NextSeq 500 high-throughput Illumina platform (2 × 150 bp paired-end, Nucleomics Core facility, KU Leuven, Belgium). Computational analysis was performed with ViPER^35^. Ambiguous bases, low-quality reads, primers, and adapter sequences were removed with Trimmomatic (v0.39). Sequences mapping to the “contaminome” (coming from the sequentiation of the four negative controls (dPBS) included in the extraction procedure) were removed using Bowtie2 (v2.4.2) in “very-sensitive” mode^36^. Quality-controlled reads were de novo assembled into a set of contigs using MetaSPAdes (v3.15.3) using 21,33,55 and 77 k-mer length^37^. A set of non-redundant scaffolds was obtained by clustering the contigs with a length greater than 500 bp at 95% average nucleotide identity and 85% coverage using CheckV’s clustering scripts^38^. Instead of calculating abundances by mapping the quality-filtered reads to the complete set of non-redundant scaffolds, reads were only mapped against the representatives of the cluster containing a scaffold from that sample to avoid false-positive detection of closely related sequences. Abundances per sample were obtained by mapping the quality-controlled reads back to the set of representative scaffolds using bwa-mem2 (v2.2.1)^39^. Representative scaffolds with a horizontal coverage of 70% or higher were kept for further analyses.

### Eukaryotic viruses

Eukaryotic viruses were identified and classified by homology-based approaches. The representative scaffolds set was compared against the NCBI nucleotide database using BLASTN (v2.11.0, e-value ≤ 1e−10)^40^, and against a non-redundant protein sequence database using DIAMOND (v.2.0.13, sensitive mode)^41^ (nonredundant [nr] and nucleotide [nt] databases downloaded from NCBI) and CAT (v5.2.3)^42^. Classification was based on the principle of lowest common ancestor as determined by the ktClassifyBLAST module in KronaTools (v2.8)^43^.

### Prokaryotic viruses

Prokaryotic viruses (bacteriophages) were identified using Virsorter2 (v2.2.3) with score ≥ 0.5^44^. The completeness of these contigs was determined with CheckV (v0.8.1)^38^. Bacteriophages were selected for further analysis based on a combination of Virsorter2 identification and ≥ 50% completeness. Classification was performed as described in the previous section. Additionally, taxonomic classification was expanded using marker gene approaches as determined by Cenote-Taker2 (v2.1.3)^45^. The lifestyle of bacteriophages was determined based on the appearance of lysogeny-specific genes. These genes were predicted using the functional annotation module of Cenote-Taker2 and can be found in Supplementary Table 2^45^. The host of bacteriophages was determined using Random Forest Assignments of Hosts (RaFAH, v0.3). Bacterial hosts were predicted on phylum level with score ≥ 0.14^46^.

### Statistical analysis

Results are presented as mean ± standard error of the mean (SEM) for quantitative variables and as percentage for qualitative variables. Categorical variables were analysed using the Chi-square or Fisher’s exact test. Normal distribution of quantitative variables was checked using the Shapiro–Wilk test. Comparison between two groups were performed using unpaired t test or U-Mann Whitney depending on the normality of the data. Comparison between three or more groups were analysed using ANOVA followed by Tukey post-hoc regardless the normality of the data. P values < 0.05 and false discovery rates (FDRs) < 0.05 were considered as statistically significant. Statistical analysis was performed using GraphPad Prism 8 (GraphPad Prism^®^, La Jolla, California, USA), R software (version 4.0.5) and R Studio (version 1.4.1105).

Alpha and beta diversity were analysed using phyloseq^47^: α-diversity is a measure of sample-level species richness, whereas β-diversity describes inter-subject similarity of microbial composition and facilitates the identification of broad differences between samples. The measure of α-diversity was analysed using *Observed Features*, *Fisher’s alpha*, *Shannon index*, *Simpson index*, and *Pielou’s index* with the plot_anova_diversity function of the microbiomeSeq package. *Observed Features* and *Fisher’s alpha* are based in richness, *Pielou’s index* is based in evenness and *Shannon index* and *Simpson index* are based in diversity (richness + evenness). The measure of β-diversity was analysed using Bray Curtis (ordinate function from phyloseq package) and visualized using Principate Coordinate Analysis (PCoA) (plot_ordination function from phyloseq package) and statistically significant differences were evaluated with PERMANOVA (adonis2 and pairwise.adonis functions from the vegan package). Finally, the analysis of the differential composition of microbiomes was carried out with DESeq2 at phylum and class taxonomic levels regarding phages and at phylum, family, and genus taxonomic levels regarding eukaryotic viruses. When required, statistical significance was evaluated with the adequate non-parametric test. All these analyses were performed using R (v3.6.3) and R Studio (v1.2.1335).

### Compliance with ethics guidelines

This study was performed following the Helsinki Declaration and was approved by the Committee for Ethics in Drug Research in La Rioja (CEImLAR) (28 February 2019, reference number 349). All participants provided their written informed consent.

## Results

### Clinical and demographical characteristics of participants

Supplementary Table 3 shows the main characteristics of the recruited population. Viral load of naive patients was 622,252.9 ± 341,039.3 copies/ml, whereas INSTIs-treated patients showed indetectable viral load (< 20 copies/ml), as expected. The average time under treatment of the INSTIs-treated patients was 33.27 ± 5.04 months. Statistically significant differences were observed between the naive group and INSTIs-treated patients in terms of CD4 levels (p < 0.01) and CD4/CD8 ratio, both higher in the INSTIs-treated group. However, no statistically significant differences were observed between the naive group and INSTIs-treated group patients in nadir CD4 levels. Statistically significant differences were observed between the control and the naive group in terms of gender (p < 0.01), age (p < 0.05), systolic blood pressure (p < 0.05), diastolic blood pressure (p < 0.05), and smoking habits (p < 0.05). Thus, males were less represented in the control group (34.62%) in contrast to HIV-infected patients (80% and 86.67% in naive and INSTIs-treated patients, respectively). Mean age of the control group was higher than that observed in the naive group. However, these differences disappeared when comparing the controls against the INSTIs-treated group. No statistical differences on age were observed among the naive and INSTIs-treated group. Both systolic and diastolic blood pressure were higher in the naive group compared to the control group. No differences were observed when the controls were compared to INSTIs-treated patients and among both HIV-infected groups. Of note, none of the naive HIV-infected patients referred hypertension. Thus, these differences on blood pressure could be explained as “white coat hypertension” generated in those patients that have received the news of being HIV-positive. Smoking habits were also higher in the naive group and in INSTIs-treated group compared to the control group (11.54% *vs.* 46.67% and 66.67%, respectively). Moreover, no differences were observed neither in the mode of transmission, nor in AIDS events (only one naive patient with AIDS because of a late diagnosis), nor in the coinfection with HCV or HBV among both HIV groups (only two INSTIs-treated patients presented confection with HCV with a very low grade of fibrosis: F0/F1).

### Reads and contigs distribution

**Figure 1:**
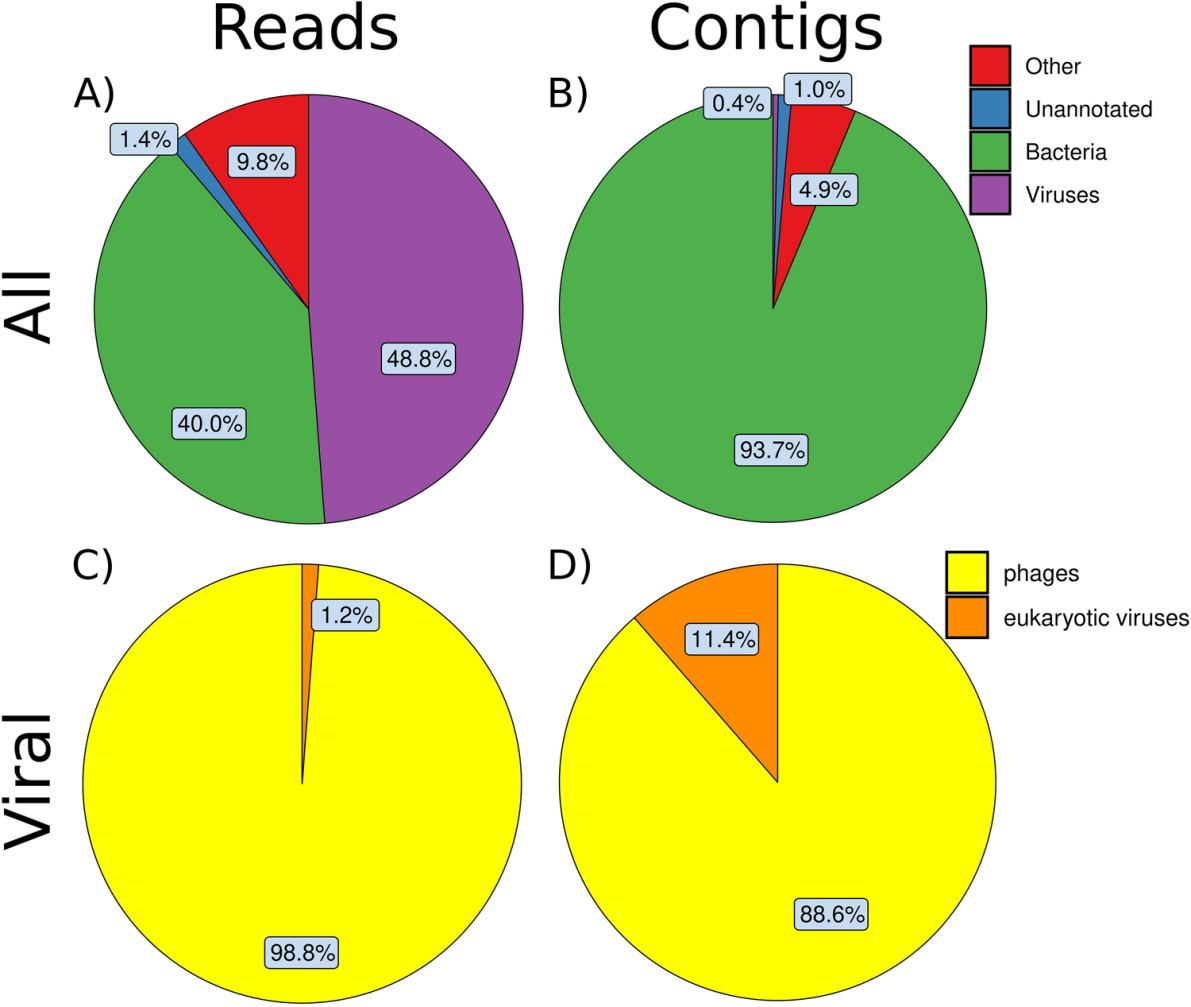
Percentage of the reads (**A,C**) and the contigs (**B,D**) that correspond to different superkingdoms (**A,B**) and viral categories (**C,D**).

After the sequencing and the quality control, 48.8% of the reads (99,038,911 reads) mapped to viruses (Fig. 1A) and, of them, 98.8% belonged to phages and 1.2% to eukaryotic virus (Fig. 1C). Among the rest of the reads, 40% mapped to bacteria, 9.8% to other microorganisms and only 1.4% were unannotated (Fig. 1A). On the other hand, only 0.4% of the contigs mapped to viruses (Fig. 1B) and, of them, 88.6% belonged to phages and 11.4% to eukaryotic viruses (Fig. 1D). Among the rest of the contigs, 93.7% belonged to bacteria, 4.9% to other and 1% to unannotated (Fig. 1B).

### Eukaryotic virus diversity and composition

#### Alpha and beta diversity of eukaryotic viruses

The analysis of the α-diversity of eukaryotic viruses did not show statistically significant differences in the indexes analysed (*Observed features*, *Simpson index*, *Shannon index,* and *Pielou’s evenness*) (Supplementary A). However, a slightly decreasing tendency in *Observed features*, *Simpson index,* and *Shannon index* in the naive group compared to the controls was observed, which seems to be reversed after INSTIs-based treatment, although without statistical significance. In the same line as α-diversity, the analysis of β-diversity did not reveal a different clustering between the three groups (Supplementary Fig. 2B).

#### Distribution of eukaryotic viruses

Eukaryotic viruses were classified into three groups: animal infecting viruses (4 families), plant and fungi infecting viruses (9 families) and small circular viruses (7 families). Supplementary Fig. 2C shows the presence of these viruses and their relative abundance in controls/uninfected subjects compared to naive and INSTIs-treated groups. Our data revealed that plant and fungal viruses were the most prevalent viruses, with members of the *Virgaviridae* family being the most abundant, followed by small circular viruses. Although plant viruses are likely passengers, they were the only group found in more than 50% of all samples. On the other hand, animal infecting viruses were the least abundant. Due to the low percentage of reads belonging to eukaryotic viruses compared to those belonging to phages and the wide deviation between samples, DESeq analysis did not reveal differentially abundant eukaryotic viruses among the three groups.

### Phage diversity and composition

#### Alpha diversity of phages

**Figure 2:**
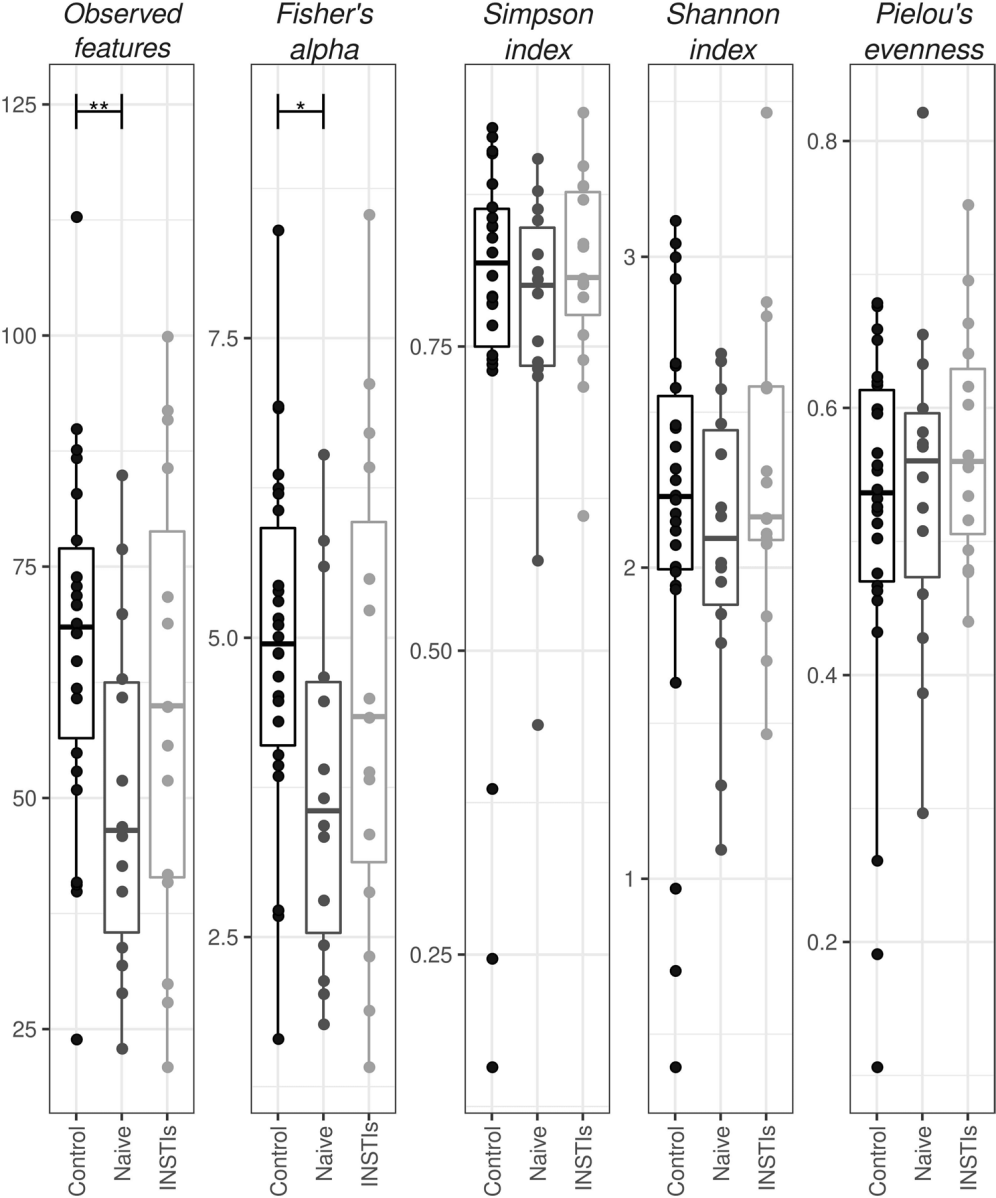
Different indexes of α-diversity from phages in faecal samples of the studied population. *p < 0.05 vs. control, **p < 0.01 vs*.* control. INSTIs (integrase strand transfer inhibitors-based treatment).

A significant decrease in *Observed features* and *Fisher’s alpha* indexes was observed in HIV-naive patients compared to controls (p < 0.01 and p < 0.05 respectively). Such decrease was not present in INSTIs-treated group when compared to controls (Fig. 2). No differences were observed either in *Simpson index, Shannon index* or in *Pielou’s evenness*.

#### Beta diversity of phages

**Figure 3:**
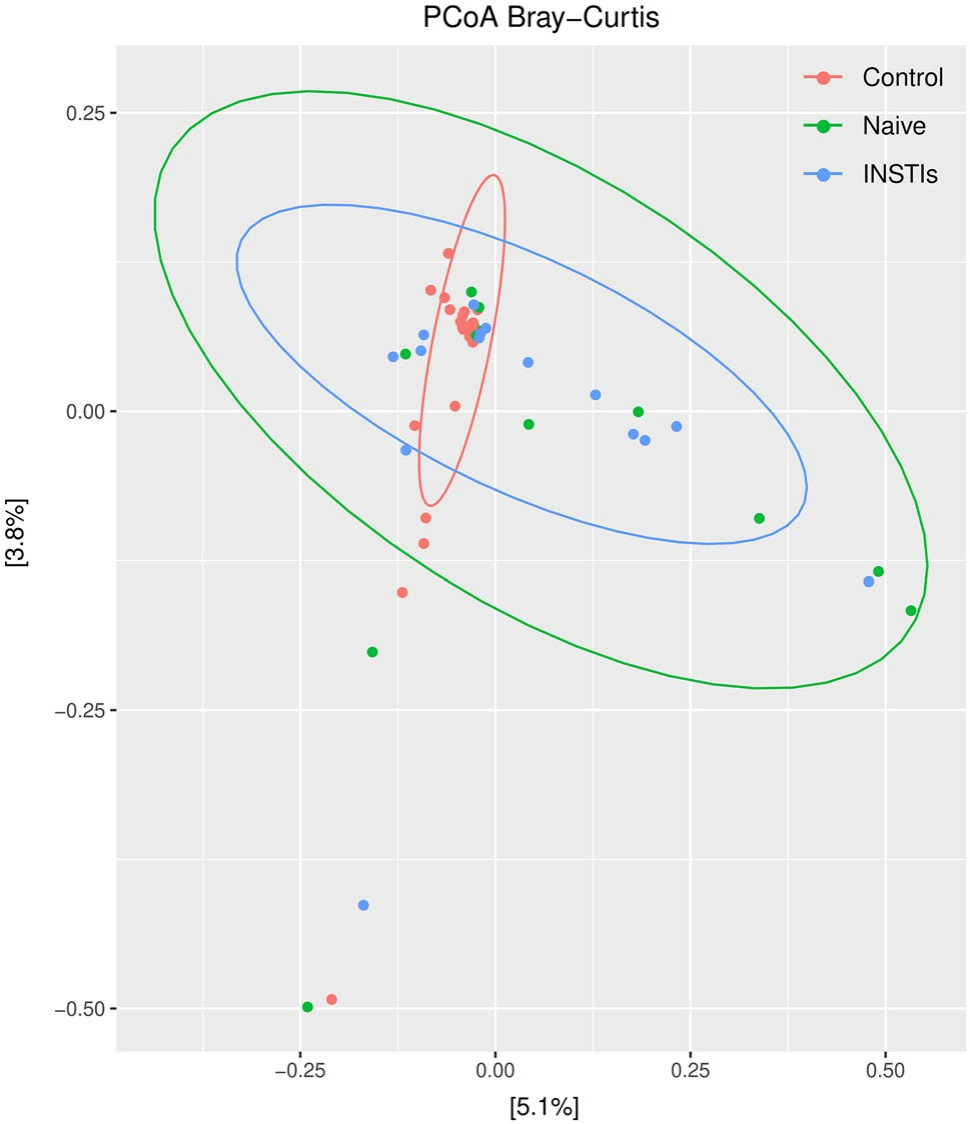
PCoA from phages in faecal samples from the studied population [accounting for 8.9% of the total variation (Component 1 = 5.1% and Component 2 = 3.8%)]. Results are plotted according to the first two principal components. Each circle represents a sample: red circles represent the uninfected volunteers, green circles represent the naive group, and blue circles represent the INSTIs-treated group. The clustering of samples is represented by their respective 95% confidence interval ellipse. padj < 0.01 naive vs. control and padj < 0.05 INSTIs vs*.* control. INSTIs (integrase strand transfer inhibitors-based treatment).

Figure 3 shows the PCoA obtained from the studied population. The control group is clearly different from the naive group (padj < 0.01) and the INSTIs-treated group (padj < 0.05). However, statistically significant differences were not observed between the naive group and the INSTIs-treated patients in terms of β-diversity, although it is worth mentioning that samples coming from INSTIs-treated patients grouped more closely together compared to those of naive patients, as can be observed in Fig. 3.

#### Differential abundance of phages

DESeq analysis revealed a statistically significant higher abundance of members of the *Caudoviricetes* class in naive patients compared to the non-infected group and a statistically significant lower abundance of the members of the *Malgrandaviricetes* class in INSTIs-treated patients compared to the control group. However, differences between naive and INSTIs-treated patients were not detected (Table 1).

**Table 1:** Phage taxonomical ranks which present a differential abundance in the studied population.

Control vs.							
Naive				INSTIs			
Category	Taxonomic group		padj	Category	Taxonomic group		padj
Phylum	Uroviricota	↑	0.011	Phylum	Phixviricota	↓	< 0.001
Class	Caudoviricetes	↑	0.011	Class	Malgrandaviricetes	↓	< 0.001

*INSTIs* integrase strand transfer inhibitors-based treatment.

#### Lifecycle prediction of phages

**Figure 4:**
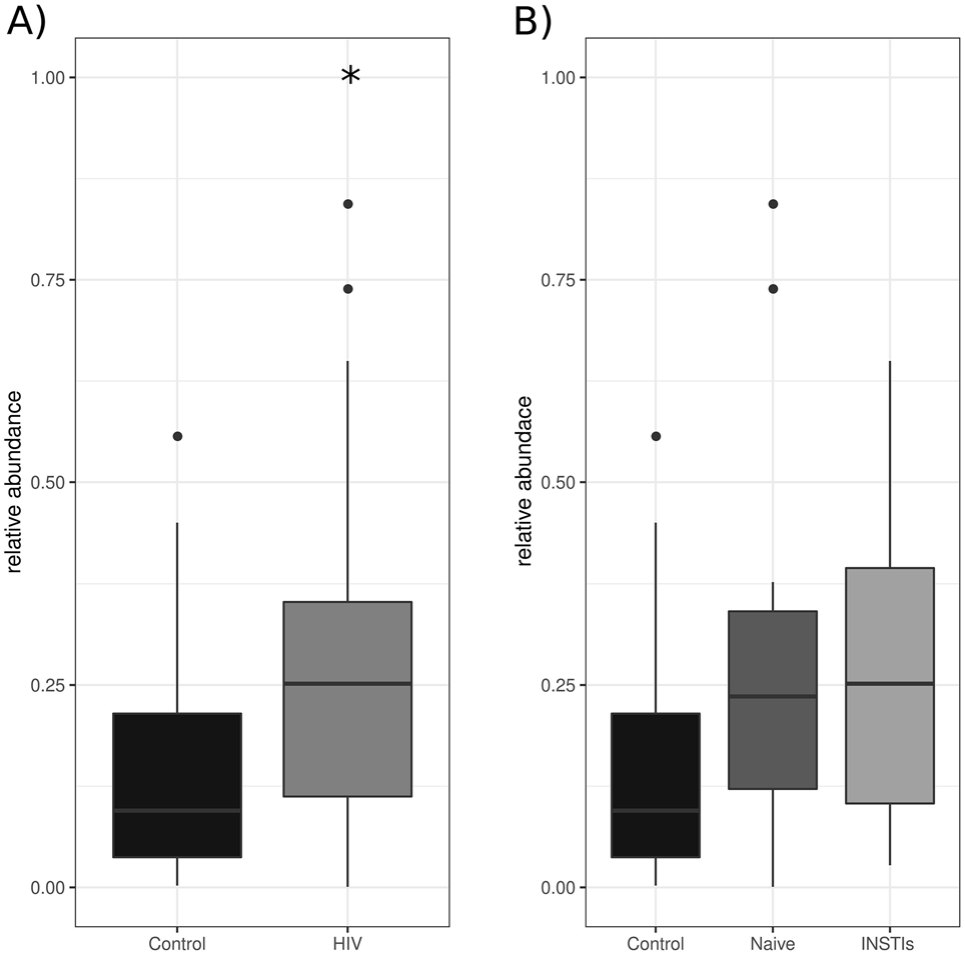
Relative abundance of the lysogenic phages comparing control group vs. HIV-infected patients (**A**) and control group vs. naive patients vs. INSTIs-treated patients (**B**). *p < 0.05 vs. control. HIV (human immunodeficiency virus), INSTIs (integrase strand transfer inhibitors-based treatment).

Comparison between controls and HIV-infected groups revealed a statistically significant increase in the relative abundance of lysogenic phages in HIV-infected patients (p < 0.05 *vs.* control) (Fig. 4A), that was not reversed after INSTIs-based treatment (Fig. 4B). These differences could not be solely explained by changes in the relative abundance of *Caudoviricetes* class (Supplementary Fig. 3).

#### Host prediction of phages

**Figure 5:**
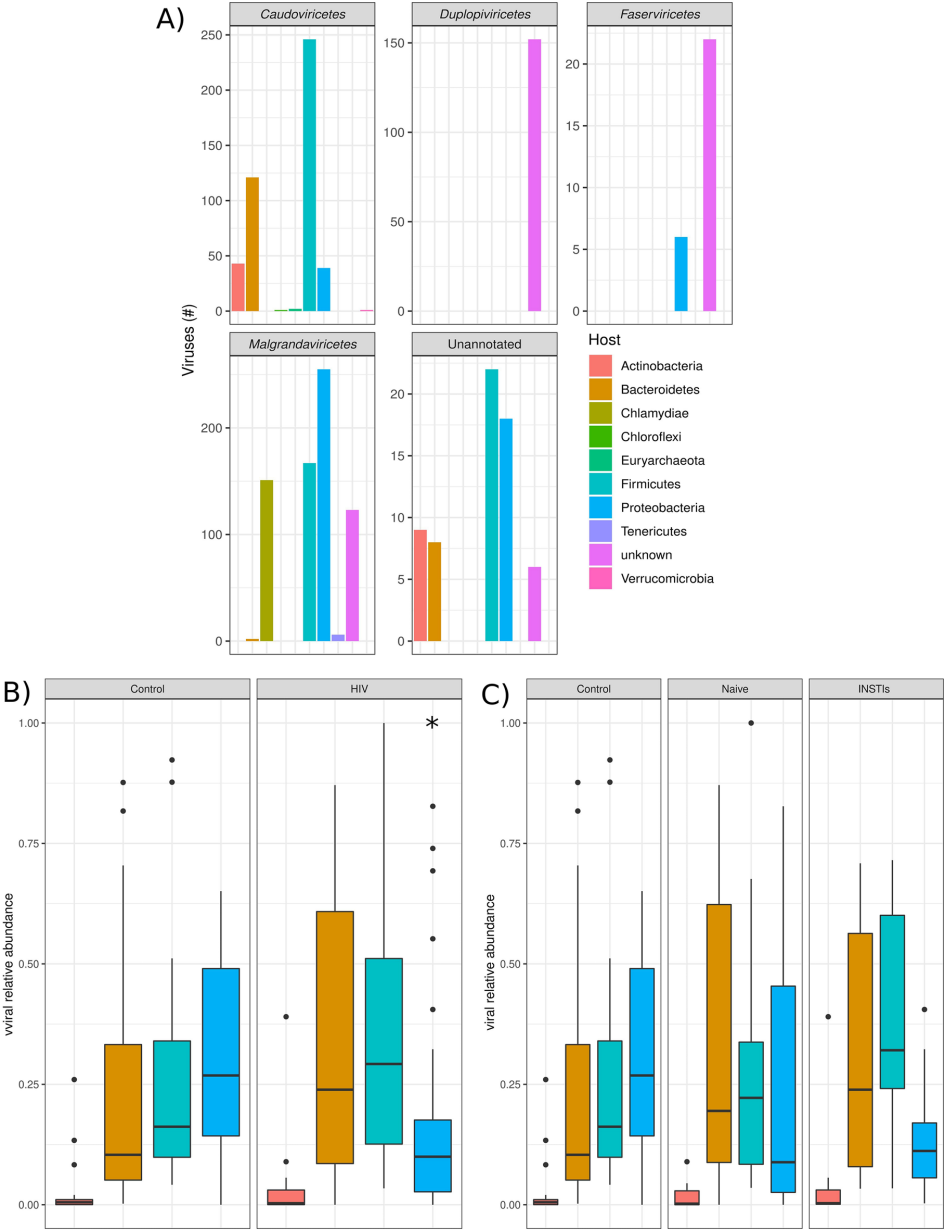
Host prediction of the phages belonging to different classes. (**A**) Prediction of which bacteria is infected by phage grouped based on class-level taxonomy. (**B**) Differences between control and HIV-infected patients in the relative abundance of the phages infecting bacterial phyla. (**C**) Differences between control, naive, and INSTIs in the relative abundance of the phages infecting bacterial phyla. *p < 0.05 vs. control. INSTIs (integrase strand transfer inhibitors-based treatment).

Figure 5A shows the host prediction of the four classes of phages detected (*Caudoviricetes*, *Duplopiviricetes*, *Faserviricetes,* and *Malgrandaviricetes*) and of those viruses which were unable to be classified (Unannotated). *Caudoviricetes* were predicted to mainly infect members of the phyla Firmicutes, followed by Bacteroidetes. *Malgrandaviricetes* were predicted to mainly infect members of the Proteobacteria phylum followed by Firmicutes and Chloroflexi phyla. The main host of *Duplopiviricetes* and *Faserviricetes* could not be detected and, therefore, was identified as “unknown”.

Finally, the comparison between the relative abundance of the phages infecting each bacterial phylum revealed that INSTIs-based treatments were not able to restore the decrease observed in the relative abundance of Proteobacteria-infecting phages (p < 0.05 HIV vs. control) (Fig. 5B,C).

## Discussion

To our knowledge, this is the first time that DNA and RNA viruses have been studied in the gut of HIV-infected people with and without treatment. In addition, the impact of INSTIs-based treatments on the gut virome composition of HIV-infected people has also been deeply analyzed. Therefore, this study provides a first and preliminary “snapshot” that describes what is happening in gut virome after HIV infection and INSTI-treatment. These findings constitute a very interesting and novel step in completing our knowledge of the impact of HIV and INSTIs on gut, and, in general terms, in the long-term consequences of HIV infection and ART treatment.

Our study revealed that in our gut samples (stools), most of the viral reads and contigs belonged to phages while eukaryotic viruses were less abundant, which is in line with previous studies that highlight that bacteriophages are the vast majority of the viral component in the human gut^48,49^. In this context, the lack of differences in alpha and beta diversity of eukaryotic viruses could be due to the low number or reads obtained belonging to these viruses. However, a slightly non-significant tendency of a decreased richness and diversity in naive patients compared to the controls was observed, along with a partial recovery after INSTIs-based treatments. Besides, our results (Supplementary Fig. 2C) revealed that plant and fungal infecting viruses (mainly members of the *Virgaviridae*) were the most abundant followed by small circular viruses and animal infecting viruses suggesting that most of the eukaryotic viruses are diet-derived. These results differ from those obtained by Monaco et al*.* (2016)^24^ who reported that *Adenoviridae*, *Anelloviridae*, *Circoviridae,* and *Papillomaviridae* viruses, all of them animal infecting viruses, were the most abundant eukaryotic viruses in the enteric virome of HIV-infected subjects. However, their study only analyzed DNA viruses, so their viral community is quite different from ours, in which we have included both DNA and RNA viruses. Besides, the HIV cohort analyzed in Monaco´s study was different from ours in terms of lifestyle, diet, age, ART, and treatment duration (33.27 ± 5.04 months vs. 6.7 years).

Regarding bacteriophages, a significant decrease in *Observed features* and *Fisher’s alpha* indexes was observed in naive patients compared to uninfected controls. However, INSTIs-based treatment was able to reverse such decrease, since no significant differences were observed among treated patients and the control group. These results are contrary to those obtained by Monaco et al*.*^24^*,* who reported no significant differences in richness or *Shannon* diversity of bacteriophage families or genera by HIV infection or treatment status. The differences among both studies could be due to several factors associated with the population recruited and described before and by the fact that only DNA viruses were analyzed in the study of Monaco et al.^24^ whereas we have included both DNA and RNA viruses. In fact, phages can be DNA and RNA viruses, therefore, our study is closer to reality because it assesses the viral community as a whole. However, another very important factor (often overlooked) could be the difference in the bioinformatics approaches carried out. In fact, in our study we have used several recently developed state-of-the-art phage analyses tools (described in Methods section). This might also explain the identification of a different clustering pattern of HIV-infected patients considering β-diversity that has not been shown before.

Our study has also showed a statistically significant higher abundance in *Caudoviricetes* class in naive patients compared to the control group. *Caudoviricetes* are dsDNA phages that have been revealed to be increased in IBD^50^. Therefore, the higher abundance observed in *Caudoviricetes* class might be associated to, or be responsible for the increased inflammatory state and gut permeability (measured through fecal calprotectin levels) observed in HIV-infected patients that is abolished after INSTI-based treatment^29^. Thus, the role of this viral class deserves more attention in the context of HIV infection, inflammation, and gut permeability. On the other hand, we observed a statistically significant decrease in members of the *Malgrandaviricetes* class (containing circoviruses) in INSTIs-treated patients, although, —up to date—their physiological meaning is unknown. Thus, more studies are needed in order to determine the role of this phage class in the gut in the context of HIV infection and INSTIs-based treatment.

Interestingly, we observed an increase in lysogenic phages related to HIV infection that was not reversed by INSTIs-based treatments. Our hypothesis is that inflammation triggered by HIV infection creates a stressful state in the enteric environment that, by one side reduces bacteriome α-diversity^29^; and, on the other side, induces a selection in phages towards lysogenic profiles since lytic virions have more difficulties to find a new bacterial host to infect. In addition, these effects were not reversed by INSTIs-based treatments, so more studies are required to analyse the possible clinical implications of such findings.

We have also revealed for the first time a significant decrease in Proteobacteria-infecting phages in HIV-infected patients, which was not restored by INSTIs-based regimens. This decrease could be triggered by the statistically lower abundance of phages belonging to *Malgrandaviricetes* class (which mainly infect members of the phylum Proteobacteria) revealed in the INSTIs-treated group (Fig. 5A). These results are very interesting because they correlate with the increase observed in some Proteobacteria taxonomical orders (specifically Aeromonadales order and *Succinivibrio genus*) in HIV-infected patients^29^. These associations among bacteriome and virome are even noteworthy since are based on the same individuals (and same samples). Furthermore, taking into account the increase observed in some Proteobacteria taxonomical orders in HIV-infected patients and its possible relationship with the decrease revealed in Proteobacteria-infecting phages in HIV-infected patients (Fig. 5B) and considering that previous studies suggests that *Succinivibrio* could be associated with defects in gastrointestinal functions, such as diarrhoea and abdominal pain^51–54^, a phage therapy focused on decreasing *Succinivibrio* presence could be an improvement in quality of life of these patients.

This study certainly has some limitations. Some of the patients were recruited during COVID pandemic. However, none of them reported symptoms related to COVID before the date of sample collection, they were not vaccinated in the previous month and reads belonging to *Coronaviridae* family were not detected in any of the samples, so the effects observed can be attributed only to HIV infection and ART status. Besides, the number of patients is small (what increases the probability of type-II error), but it was enough to detect differences between the groups as in other studies^18,19^. Moreover, there are some differences between the control group and the HIV-infected groups, such as gender, age, and smoking habits, all of them factors that could have an impact on GM. However, the two HIV-infected groups were well-balanced taking into account these factors, so the differences observed between them will be independent from these factors and could be attributed to INSTIs-based treatments. On the other hand, when the control group is compared with all HIV-infected patients (both naive and INSTIs-treated together) no statistically significantly differences were observed in age, so the differences observed in the gut virome among both groups could be attributed to HIV infection. Moreover, we have performed a study comparing the control/uninfected group *vs.* naive group controlled by age (splitting into two groups, “young” and “aged”, according to the median age of the population) and only differences were observed on β-diversity, suggesting that age could be a factor which impacts gut virome composition. However, it is important to mention that since the “aged” group only included three naive patients from 19, the potential role of HIV infection more than age per se could also contribute to such actions. However, more studies with larger cohorts are needed to elucidate the impact of age on gut virome in HIV-infected people. Finally, patients fulfilled a survey focused on dietary habits/patterns to detect those habits that could have an impact on microbiota such as veganism or excessive consumptions of prebiotics and/or probiotics. None of the patients reported such habits and, on the contrary, dietary habits were quite similar among individuals. Besides, it is important to note that some of the effects observed could be attributed to the synergetic action of NRTIs used as backbone with INSTIs. However, the previous works from our group demonstrated that ARTs based on INSTIs were the ones associated with levels of systemic inflammation, sCD14 plasma levels, and microbial diversity similar to those observed in uninfected controls compared to other regimens and independently of the backbone^28,29^, which suggests that the effects reported could be mainly attributable to the INSTIs. More studies are needed in this regard.

It is worth mentioning that the lack of studies carried out in this field to compare our results could be also considered as a limitation. However, we believe that is more a strength than a limitation as we contribute to complete our knowledge of the impact of HIV and INSTIs on gut, and, in general terms, in the long-term consequences of HIV infection and INSTIs treatment.

## Conclusions

Our study suggests that HIV infection has a direct impact on gut bacteriophages and eukaryotic virus abundances, and INSTIs-based treatments (with NRTIs as backbone) were able to reverse the effects observed on α-diversity metrics. These results could serve as a first step for studying the associations between gut bacteriome and gut virome, which could allow the future development of coadjuvant therapies (based not only on probiotics/prebiotics but also phage therapies and/or fecal transplantation) to reduce bacterial dysbiosis in HIV-infected patients and, therefore, to improve the inflammatory state associated with the infection and the long-term consequences of such state. This will undoubtedly improve the health and quality of life of HIV-infected patients.

## Supplementary Information

Supplementary Information.

## Data Availability

The datasets generated during and/or analysed during the current study are available in the NCBI SRA repository, http://www.ncbi.nlm.nih.gov/bioproject/819232 and also available from corresponding author on reasonable request.
