# Supplementary material for: Impact of HIV infection and integrase strand transfer inhibitors-based treatment on the gut virome
Source: Sci Rep. 2022 Dec 15;12:21658. doi: 10.1038/s41598-022-25979-5 (PMC9755154; doi:10.1038/s41598-022-25979-5)
Supplement: Supplementary file 1 — Supplementary Information. [file 41598_2022_25979_MOESM1_ESM.docx]

**Supplementary Material for**

**Impact of HIV infection and integrase strand transfer inhibitors-based treatment on the gut virome**

Pablo Villoslada-Blanco^1^, Patricia Pérez-Matute^1§^, María Íñiguez^1^, Emma Recio-Fernández ^1^, Daan Jansen^2^, Lander De Coninck^2^, Lila Close^2^, Pilar Blanco-Navarrete^3^, Luis Metola^4^, Valvanera Ibarra^4^, Jorge Alba^4^, Jelle Matthijnssens^2^ and José A. Oteo^1,4^.

^1^Infectous Diseases, Microbiota and Metabolism Unit. Infectious Diseases Department, Center for Biomedical Research of La Rioja (CIBIR), Logroño, La Rioja, Spain.

^2^KU Leuven, Department of Microbiology, Immunology and Transplantation, Rega Institute, Laboratory of Viral Metagenomics, Leuven, Belgium.

^3^Centro de Salud Siete Infantes de Lara, Logroño, La Rioja, Spain.

^4^Infectious Diseases Department. Hospital Universitario San Pedro, Logroño, La Rioja, Spain.

^§^Corresponding author: Patricia Pérez-Matute

Infectious Diseases, Microbiota and Metabolism Unit. Infectious Diseases Department, Center for Biomedical Research of La Rioja (CIBIR), Logroño, La Rioja, Spain.

C/Piqueras 98 CIBIR building, third floor

26006 Logroño (La Rioja)

[cpperez@riojasalud.es](mailto:cpperez@riojasalud.es)

**Supplementary Table 1**

**Supplementary Table 2**

**Supplementary Table 3**

**Supplementary Figure 1**

**Supplementary Figure 2**

**Supplementary Figure 3**

**Supplementary Table 1.** INSTIs-based treatments used in HIV-infected patients.

| **INSTI** | **Backbone** | **Number of patients** |
| --- | --- | --- |
| Dolutegravir | Abacavir and lamivudine | 10/15 (66.67%) |
|  | Lamivudine | 2/15 (13.33%) |
| Bictegravir | Emtricitabine and tenofovir alafenamide | 3/15 (20.00%) |

HIV (human immunodeficiency virus infection), INSTI (integrase strand transfer inhibitor).

**Supplementary Table 2.** Predicted lysogenic-specific genes and their functions.

| **Lysogenic-specific genes** | **Function** |
| --- | --- |
| Integrase | Integrase |
| INTEGRASE | Integrase |
| Phage_integrase_family_protein | Integrase |
| SITE_SPECIFIC_RECOMBINASE_XERD | Integrase |
| Excisionase_from_transposon_Tn916 | Excisionase |
| DNA_binding_domain,_excisionase_family | Excisionase |
| Repressor_protein_CI | Repressor |
| P22_AR_repressor_domain_protein | Repressor |
| REPRESSOR_PROTEIN | Repressor |
| Cro/C1-type_HTH_DNA-binding_domain | Repressor |
| Phage_regulatory_protein_Rha_(Phage_pRha) | Repressor |
| Uncharacterized_ATPase,_putative_transposase | Transposase |
| MU TRANSPOSASE | Transposase |
| PROPHAGE_LAMBDALM01_ANTIGEN | Prophage domain |
| Mu-like_prophage_DNA_circulation_protein | Prophage domain |
| Mu-like_prophage_I_protein | Prophage domain |
| ParB protein | Lysogenic recombination |
| CHROMOSOME_PARTITIONING_PROTEIN_PARB | Lysogenic recombination |

|  | **Control** | **Naive** | **INSTIs-treated** | **p value** |
| --- | --- | --- | --- | --- |
| **Number of patients** | 26 | 15 | 15 | - |
| **Gender (men)** | 9/26 (34.62%) | 13/15 (86.67%) ** | 12/15 (80.00%) ** | **0.002** |
| **Age (years)** | 43.58±2.31 | 33.87±2.85 * | 43.67±3.39 | **0.033** |
| **BMI (kg/m**²) | 24.30±0.69 | 23.23±1.05 | 23.51±0.85 | 0.616 |
| **Waist circumference (cm)** | 85.35±2.55 | 83.83±2.86 | 85.13±2.15 | 0.916 |
| **Systolic blood pressure (mmHg)** | 120.58±2.76 | 135.67±5.67 * | 129.73±6.02 | **0.050** |
| **Diastolic blood pressure (mmHg)** | 72.19±1.94 | 81.87±3.31 * | 77.80±3.24 | **0.035** |
| **Alcohol active** | 3/26 (11.54%) | 0/15 (0.00%) | 1/15 (6.67%) | 0.578 |
| **Smoking active** | 3/26 (11.54%) | 7/15 (46.67%) * | 10/15 (66.67%) *** | **0.001** |
| **Basal CD4 (cells/µl)** | - | 464.07±76.46 | 850.53±101.68 | **0.006** |
| **Nadir CD4 (cells/ µl)** | - | 464.07±76.46 | 526.53 ± 56.30 | 0.517 |
| **CD4/CD8 ratio** | - | 0.53±0.13 | 0.84±0.10 | **0.027** |
| **Mode of transmission** | - | HS: 6/15 (40.00%) | HS: 7/15 (46.67%) | 0.716 |
|  | - | MSM: 9/15 (60.00%) | MSM: 7/15 (46.67%) |  |
|  | - | Parenteral: 0/15 (0.00%) | Parenteral: 1/15 (6.66%) |  |
| **AIDS** | - | 1/15 (6.67%) | 0/15 (0.00%) | 1 |
| **Coinfection with virus C** | - | 0/15 (0.00%) | 2/15 (13.33%) | 0.483 |
| **Coinfection with virus B** | - | 0/15 (0.00%) | 0/15 (0.00%) | 1 |

**Supplementary Table 3.** Characteristics of healthy uninfected controls and HIV-infected patients (naive and under INSTIs).

Qualitative variables are represented in percentage while quantitative variables are represented as mean ± standard error mean. P value refers to the comparation between two (naive *vs.* ART) or three (control *vs.* naive *vs.* ART) groups, as appropriate. Statistically significant p values are in bold. Asterisks indicate statistically significant differences with respect to control group (*p<0.05, **p<0.01 and ***p<0.001). AIDS (acquired immunodeficiency syndrome), BMI (body mass index), HS (heterosexual), INSTIs (integrase strand transfer inhibitors-based treatment), MSM (men who have sex with men).**
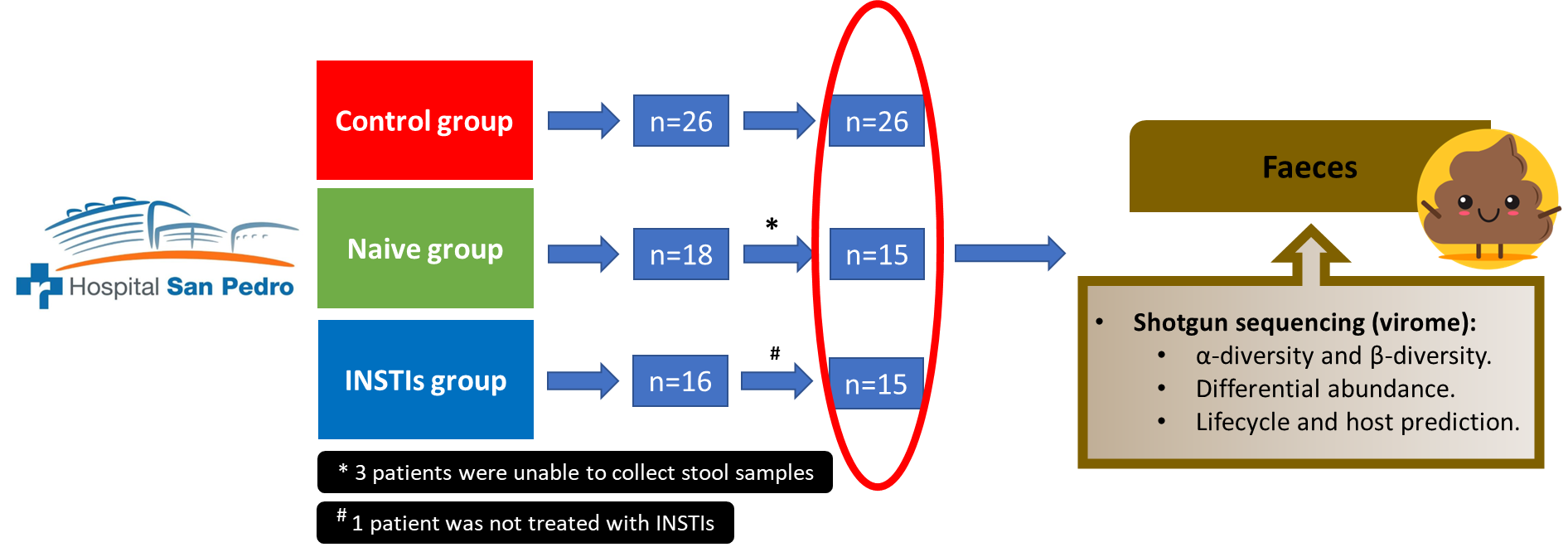
Supplementary Figure 1.** Flowchart of patient recruitment. INSTIs (integrase strand transfer inhibitors-based treatment). Original contribution.

**Supplementary Figure 2**. **A)** Different indexes of α-diversity from eukaryotic viruses in faecal samples of the studied population. **B)** PCoAs from eukaryotic viruses in faecal samples from the studied population (accounting for 23% of the total variation [Component 1 = 13.8% and Component 2 = 9.2%]). Results are plotted according to the first two principal components. Each circle represents a sample: red circles represent the uninfected volunteers, green circles represent the naive group and blue circles represent the INSTIs-treated group. The clustering of sample is represented by their respective 95% confidence interval ellipse. **C)** Heatmap of the distribution of eukaryotic viruses between control, naive and INSTIs group. The relative abundance and the presence of the different families between three groups is shown (animal infecting viruses, plant and fungi infecting viruses and small circular viruses). INSTIs (integrase strand transfer inhibitors-based treatment).


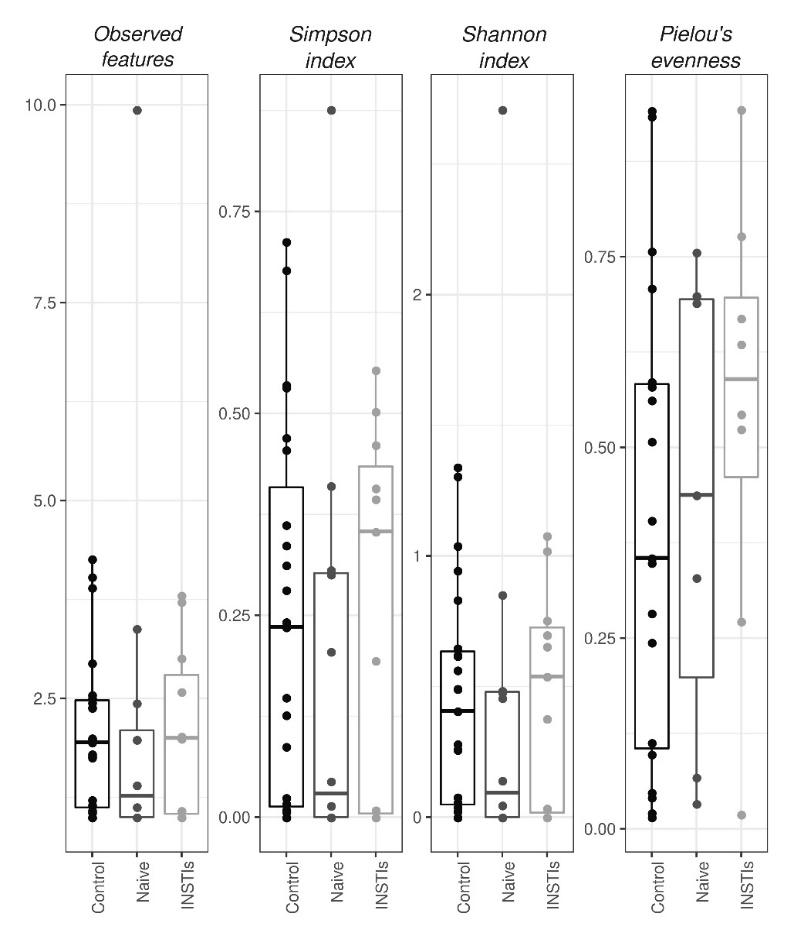

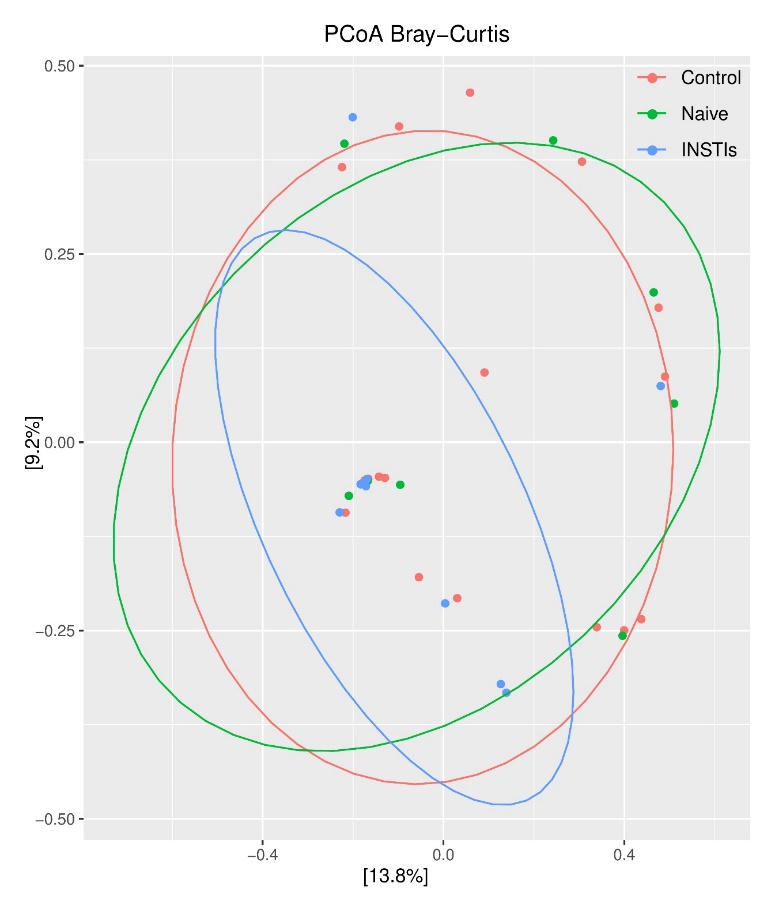

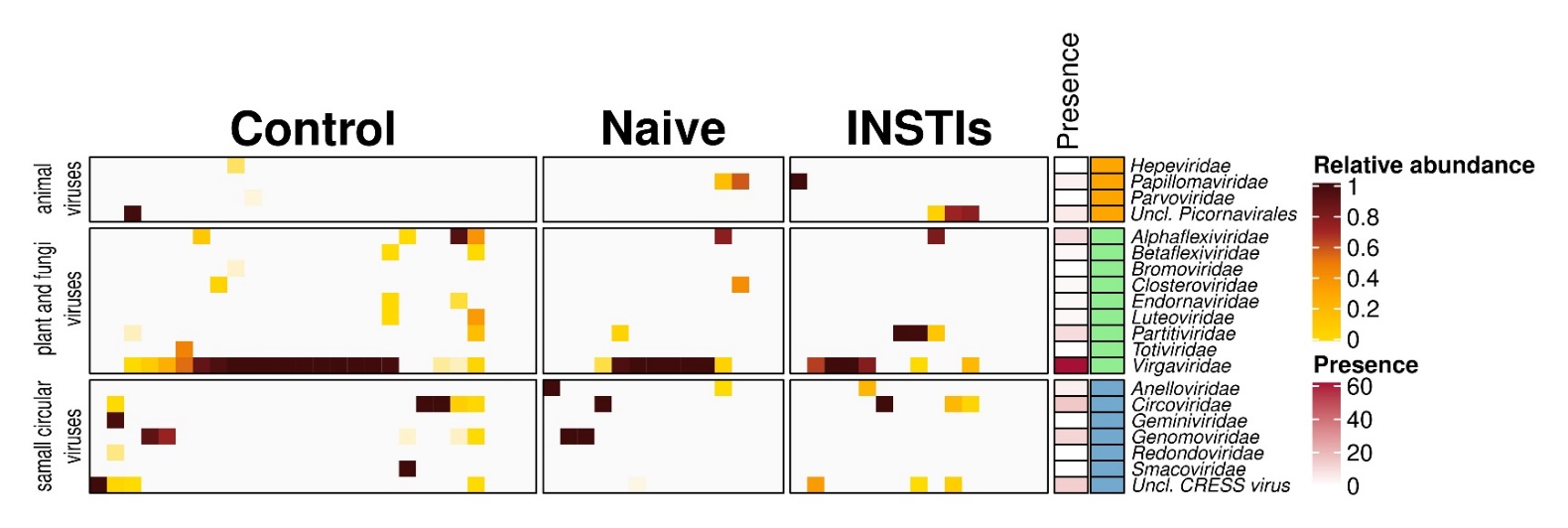


A)

B)

C)


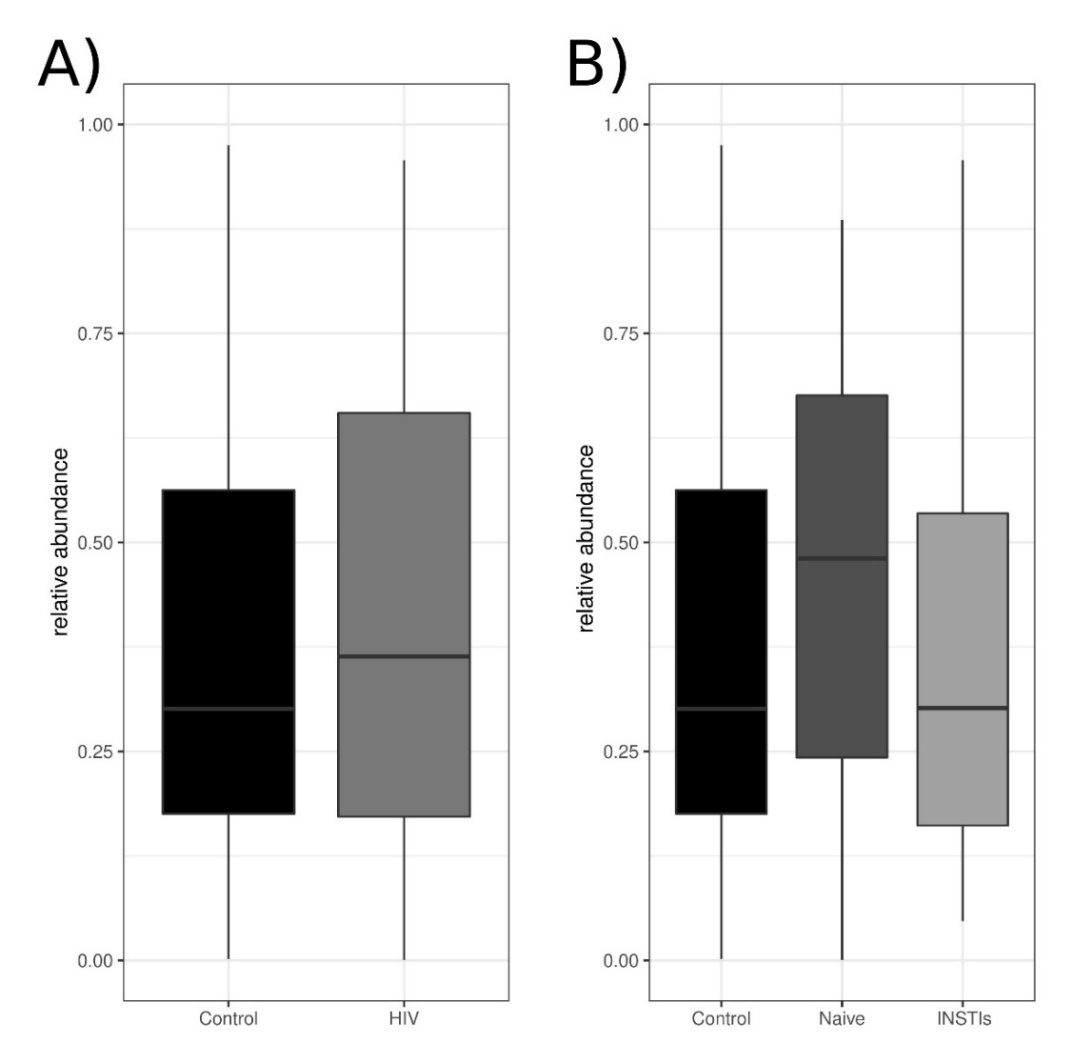
**Supplementary Figure 3.** Relative abundance of the lysogenic phages belonging to *Caudoviricetes* class comparing control group *vs.* HIV-infected patients (**A**) and control group *vs.* naive patients *vs.* INSTIs-treated patients (**B**). HIV (human immunodeficiency virus), INSTIs (integrase strand transfer inhibitors-based treatment).
